# Supplementary material for: Endogenous retroviruses co-opted as divergently transcribed regulatory elements shape the regulatory landscape of embryonic stem cells
Source: Nucleic Acids Res. 2022 Feb 15;50(4):2111–27. doi: 10.1093/nar/gkac088 (PMC8887488; doi:10.1093/nar/gkac088)
Supplement: gkac088_Supplemental_Files [file gkac088_supplemental_files.zip › Bakoulis_et_al_revision_2_supplementary_material.pdf]

Supplementary Material for:

Endogenous retroviruses co-opted as divergently transcribed regulatory elements shape the regulatory landscape of embryonic stem cells

Stylianos Bakoulis[1], Robert Krautz[1], Nicolas Alcaraz[1,2], Marco Salvatore[1], Robin Andersson[1,*]

[1]The Bioinformatics Centre, Department of Biology, University of Copenhagen, 2200, Copenhagen, Denmark
[2]Novo Nordisk Foundation Center for Protein Research (CPR), University of Copenhagen, 2200, Copenhagen, Denmark
*To whom correspondence should be addressed: robin@binf.ku.dk

Contents
Supplementary Note
Supplementary Figures 1-16

Supplementary Note:
Evaluation of multi-mapping rescue of CAGE reads

Of all mouse TE insertion events (3,677,522), only 1.3% (46,424) were associated with uniquely mapped CAGE tags in mESCs. Expressed TEs were associated with ~4% of all identified TSSs in mESCs and ~2% of uniquely mapped CAGE reads. Employing the MuMRescueLite multi-mapping rescue approach (1–3) led to an increase in the number of detected expressed TEs (the number of detected TEs increased by 77.5% to 82,383 TEs) and an overall higher expression level (Supplementary Figs. 2B, 3, 4).

Comparison of TE expression quantification after MuMRescueLite multi-mapping with an alternative quantification strategy (TELocal) (4), based on maximum likelihood alignments to annotated repeats, revealed distinct TE instances identified as expressed by each approach (Supplementary Fig. 5). The results show that MuMRescueLite and TELocal yield a comparable number of unique zero expression TE instances per family and that TE expression values for TE insertions identified by both approaches are comparable (Spearman's rho ranging between 0.53 and 0.7; Supplementary Fig. 5), in particular for ERVs. Note that TPM values are not directly comparable since TELocal is based on a predefined set of regions, while with MuMRescueLite we considered mapping to the whole genome. However, the benefit of being able to study TSSs at base pair resolution, which is not possible with TELocal that yields one expression value per locus, and to quantify the expression levels of TSSs genome-wide not having to rely on custom annotations of TE insertions made us opt for the probabilistic multi-mapping rescue (MuMRescueLite) strategy.

We further analyzed TE-associated transcription initiation events using CAGE data from exosome-depleted HeLa cells (5). We observed a similar increase in TE expression levels upon exosome depletion as observed in mESCs, and that probabilistically rescued multi-mapping reads increased the number of detected transcribed TEs (Supplementary Fig. 1).

We next investigated how rescuing multi-mapping CAGE reads affects expression level quantification of genes with TE-associated promoters in HeLa cells. Using generalized linear Poisson regression, we observed that the multi-mapping rescue approach improved the agreement between promoter-derived gene expression levels inferred from HeLa CAGE (5) with those from HeLa RNA-seq data (6) quantified from exonic reads ($p=0.0112$ and $p<2e-16$ for uniquely mapped CAGE data versus data also including rescued reads, respectively, F-test). In particular, we utilized RNA-seq expression summarized to the gene-level as the response variable of the generalized linear model and gene-level expression as measured by CAGE-seq using unique or rescued alignment approaches as continuous predictor variables, quantified as explained in the Methods. To better explore the ambiguity introduced in our CAGE dataset with the use of multi-mapping rescuing, several predictor variables of gene-level rescued CAGE expression with different window parameters (6, 20 and 50 base

pairs around each multi-mapping read) together with the CAGE expression derived from uniquely mapped reads were tested and a backward elimination approach was used to remove predictor variables with non-statistically significant p-values. We were able to identify a significant relationship both for the unique CAGE and rescued CAGE gene-level expression with a 50bp window to the RNA-seq expression, with a higher explanatory power after using the backward elimination approach (adj. R squared=0.311 for the 2-variable model versus 0.278 for the 4-variable model; lower BIC criterion for model selection=1.219621 for the 2-variable model versus 1.631852 for the 4-variable model). This shows that quantification of TE-derived transcripts with CAGE may underestimate their abundances and that rescuing multi-mapping reads with a proper window parameter around multi-mapping reads can alleviate some of these challenges.

Supplementary references

1. Faulkner,G.J., Forrest,A.R.R., Chalk,A.M., Schroder,K., Hayashizaki,Y., Carninci,P., Hume,D.A. and Grimmond,S.M. (2008) A rescue strategy for multimapping short sequence tags refines surveys of transcriptional activity by CAGE. *Genomics*, **91**, 281–288.
2. Faulkner,G.J., Kimura,Y., Daub,C.O., Wani,S., Plessy,C., Irvine,K.M., Schroder,K., Cloonan,N., Steptoe,A.L., Lassmann,T., *et al.* (2009) The regulated retrotransposon transcriptome of mammalian cells. *Nat. Genet.*, **41**, 563–571.
3. Hashimoto,T., de Hoon,M.J.L., Grimmond,S.M., Daub,C.O., Hayashizaki,Y. and Faulkner,G.J. (2009) Probabilistic resolution of multi-mapping reads in massively parallel sequencing data using MuMRescueLite. *Bioinformatics*, **25**, 2613–2614.
4. Jin,Y., Tam,O.H., Paniagua,E. and Hammell,M. (2015) TEtranscripts: a package for including transposable elements in differential expression analysis of RNA-seq datasets. *Bioinformatics*, **31**, 3593–3599.
5. Andersson,R., Refsing Andersen,P., Valen,E., Core,L.J., Bornholdt,J., Boyd,M., Heick Jensen,T. and Sandelin,A. (2014) Nuclear stability and transcriptional directionality separate functionally distinct RNA species. *Nat Comms*, **5**, 5336.
6. Andersen,P.R., Domanski,M., Kristiansen,M.S., Storvall,H., Ntini,E., Verheggen,C., Schein,A., Bunkenborg,J., Poser,I., Hallais,M., *et al.* (2013) The human cap-binding complex is functionally connected to the nuclear RNA exosome. *Nat Struct Mol Biol*, **20**, 1367–1376.

Supplementary figures

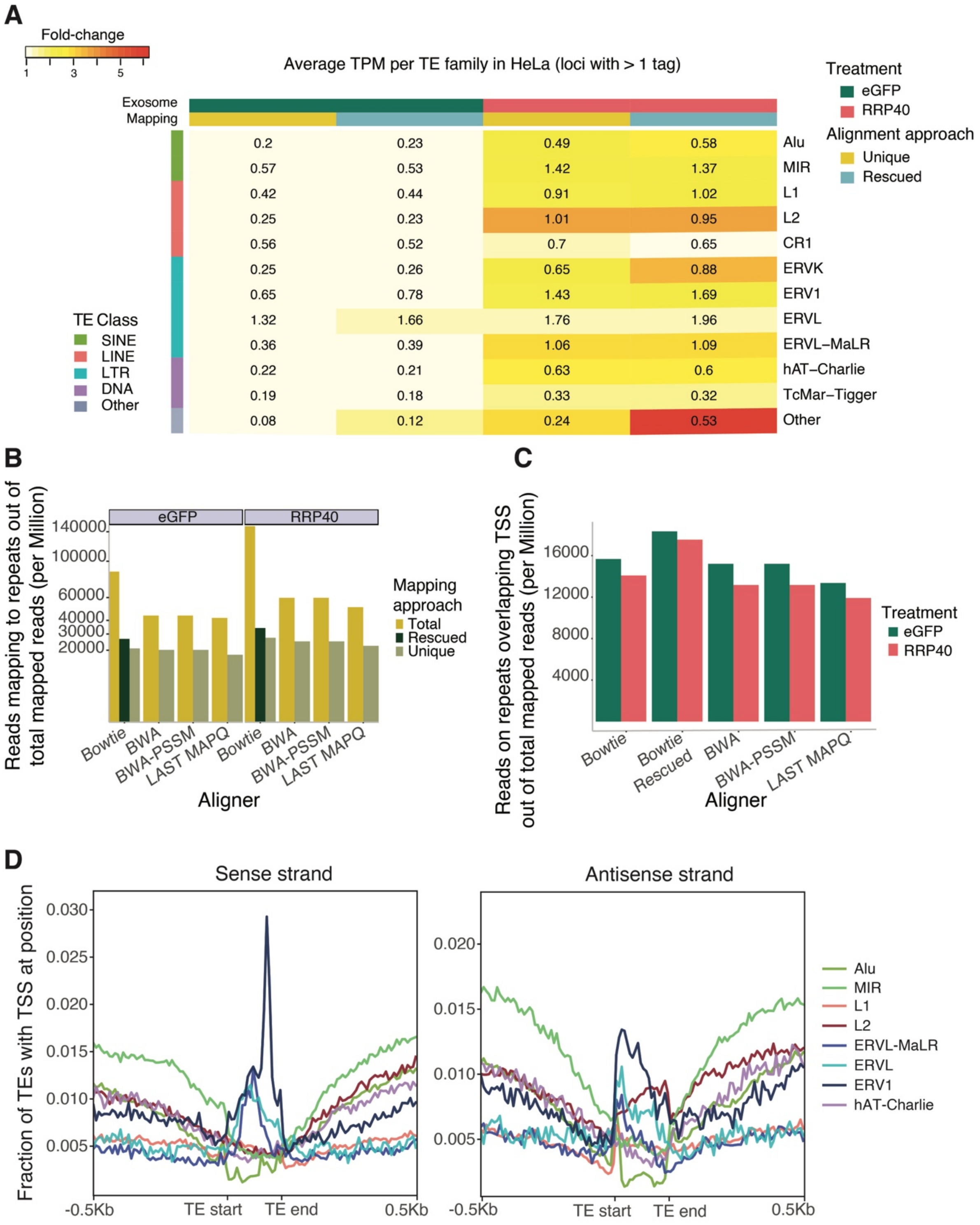

Supplementary Figure 1. A: *Heatmap of average TPM-normalized expression values quantified at the CTSS level for major TE families in HeLa S2 cells. Values for control (eGFP) or exosome-depleted (RRP40) CAGE libraries and using only uniquely mappable reads (Unique) or by employing the MumRescueLite algorithm (Rescued) are shown per column. The values in the cells are calculated as TPM values divided by the union of TEs expressed in at least one of the columns/conditions, thus representing a comparable average TPM value. The color key represents the fold-change versus the value for uniquely mappable reads in control samples.* ***B:*** *The number of CAGE tags mapping to TEs*

out of the total number of mapped reads (per million reads) using different aligners (horizontal axis) and different downstream approaches (keeping all multi-mapping reads, all uniquely mappable reads and Multimapping rescued reads with Bowtie). ***C:*** *The number of CAGE tags mapping to TEs overlapping GENCODE TSSs out of the total number of mapped reads (per million reads) using different aligners and downstream approaches (horizontal axis) as quantified in control (eGFP) and exosome-depleted (RRP40) CAGE libraries.* ***D****: Average distribution of CAGE-inferred TSS locations in HeLa S2 cells (vertical axis; expression agnostic) +/- 500 bp upstream/downstream and across the body of major TE families (horizontal axis). TSS locations are visualized separately for the sense (left panel) and antisense (right panel) strands.*

A

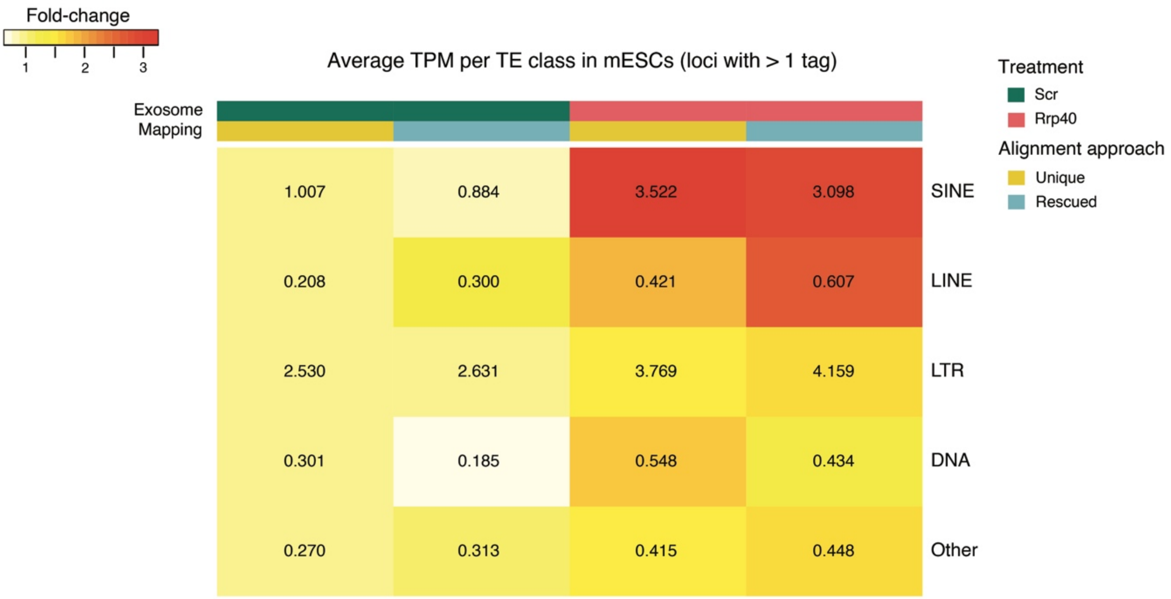

B

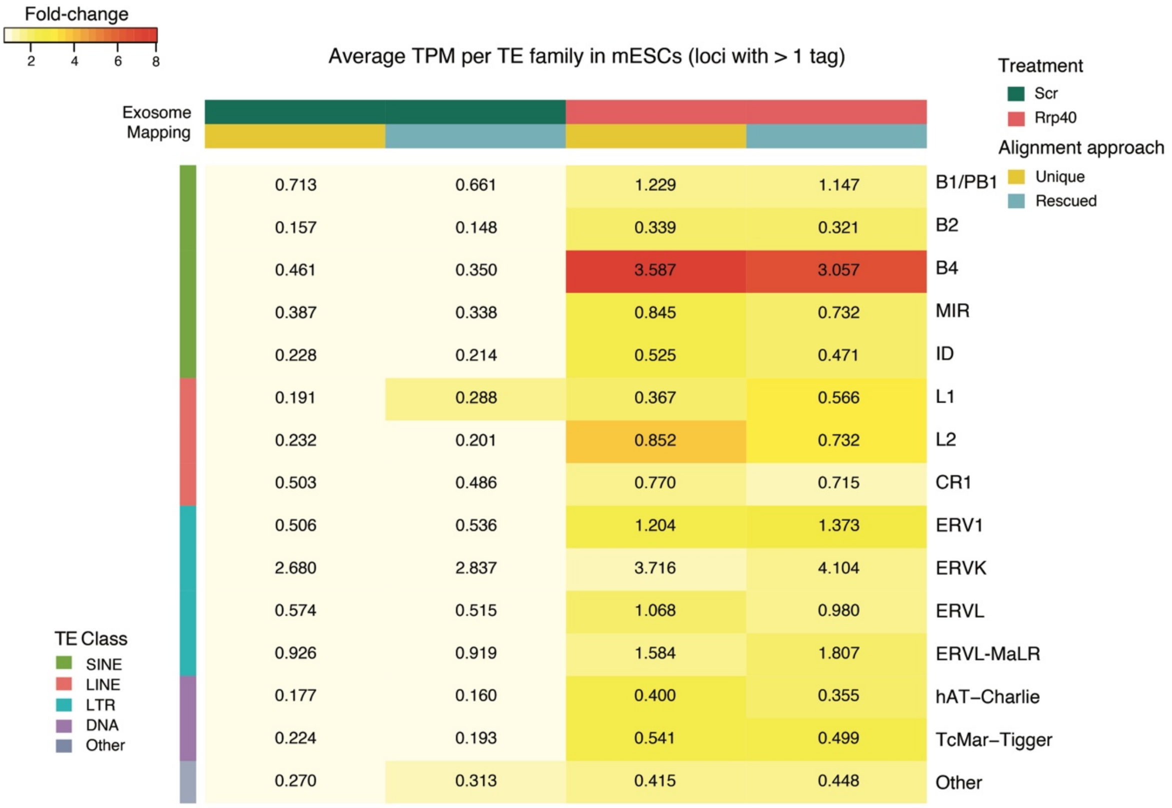

Supplementary Figure 2*. **A-B**: Heatmaps of average TPM-normalized expression values quantified at the CTSS level for major TE classes (**A**) and major TE families (**B**) in mESCs. Values for control (Scr) or exosome-depleted (Rrp40) CAGE libraries and using only uniquely mappable reads (Unique) or employing the MumRescueLite algorithm (Rescued) are shown per column. The values in the cells are calculated as TPM values divided by the union of TEs expressed in at least one of the columns/conditions, thus representing a comparable average TPM value. The color key represents the fold-change versus the value for uniquely mappable reads in control samples.*

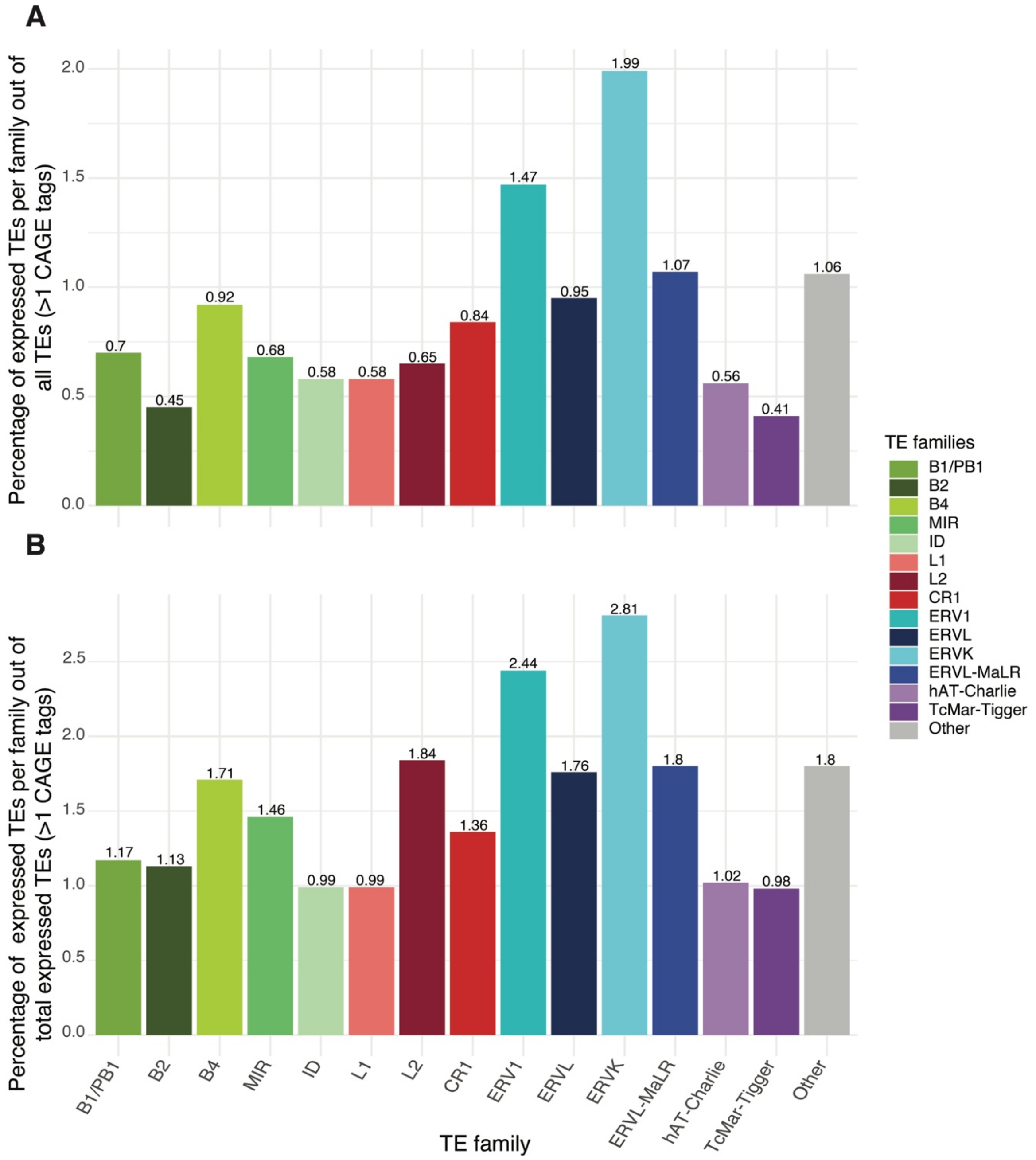

***Supplementary Figure 3*. A-B:** *Percentage of transcribed TEs at the TE family level out of all TE insertions annotated in RepeatMasker (A) and out of all identified TEs with two or more CAGE tags (B).*

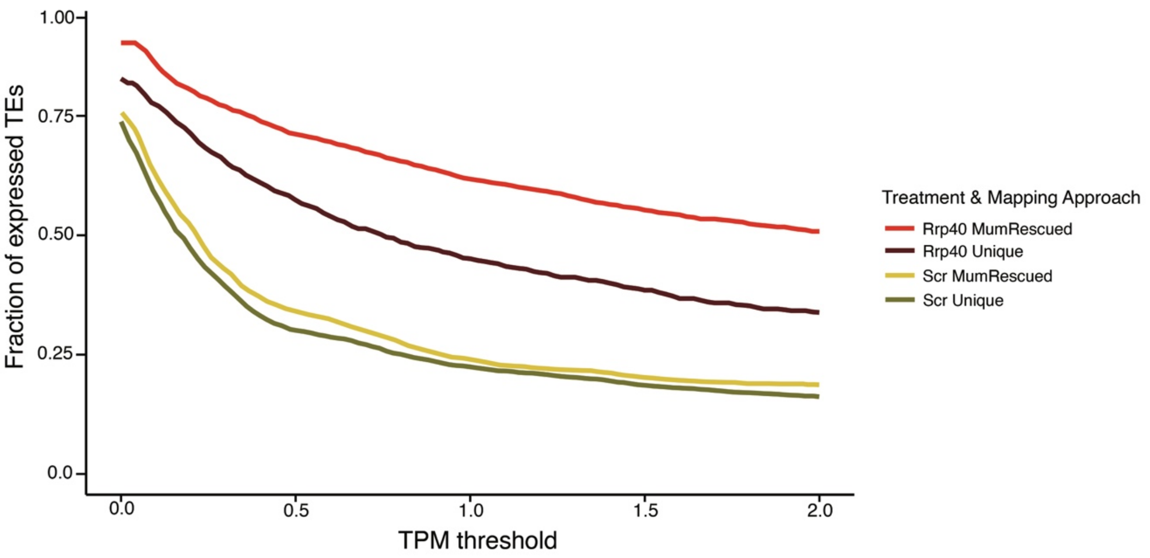

Supplementary Figure 4. *Fraction of expressed TE insertions versus TPM expression threshold (horizontal axis) over all expressed TEs in control (Scr) and exosome-depleted (Rrp40) libraries, counting only uniquely mappable (Unique) or, in addition, also rescued (MumRescueLite-processed) reads.*

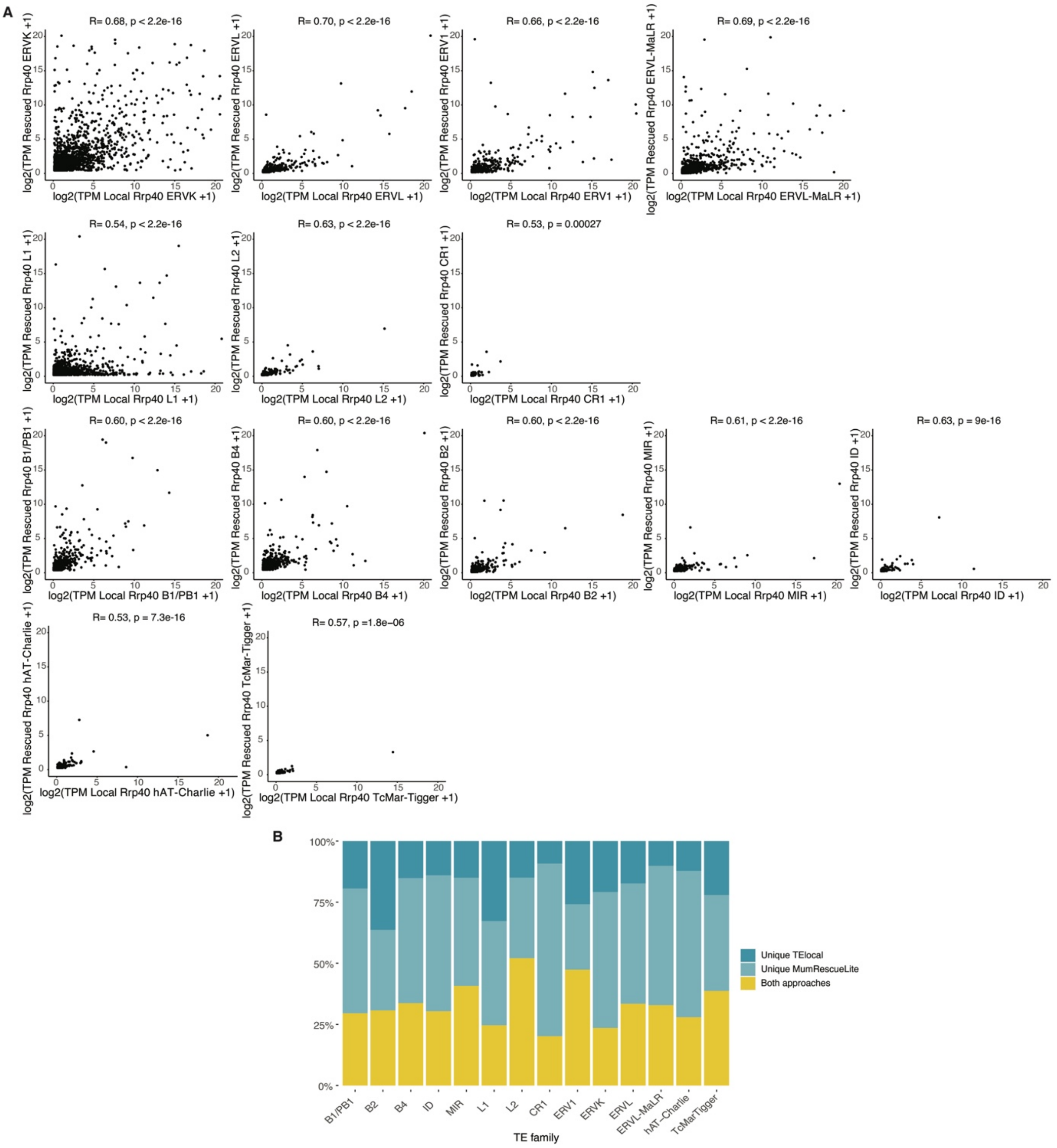

***Supplementary Figure 5**. **A**: Quantified expression levels (log$_2$-transformed TPM-normalized counts) by MumRescueLite (vertical axis) and TElocal (horizontal axis) from pooled CAGE libraries. Expressed TE insertions detected by both approaches for major TE families are shown. **B**: Proportions of expressed TEs identified by TElocal, MumRescueLite or both approaches for major TE families.*

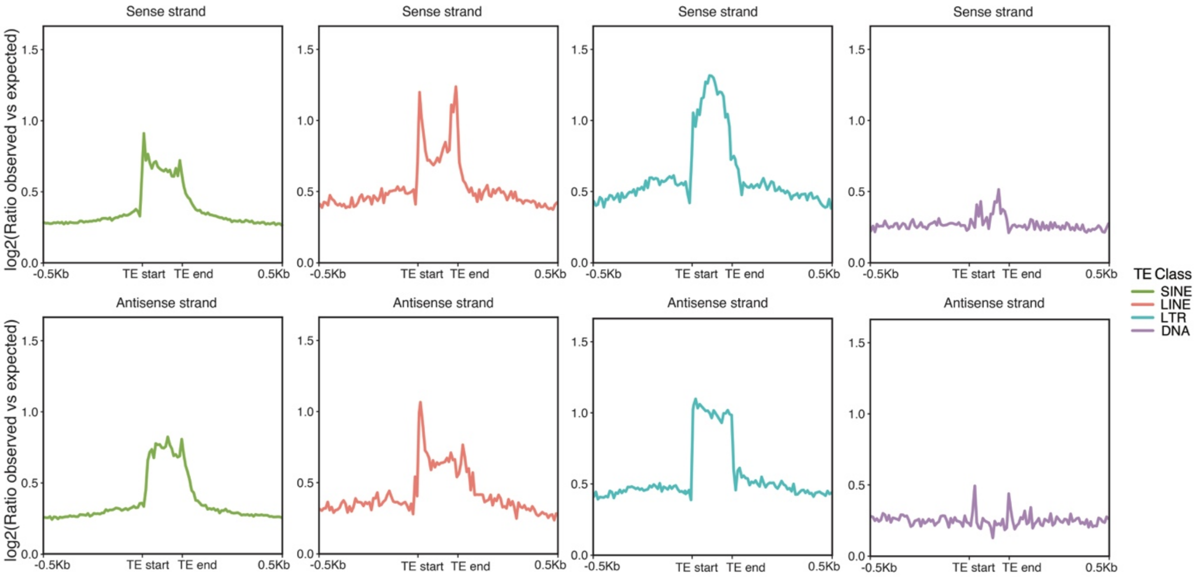

Supplementary Figure 6*. Log$_2$ ratio of average observed versus expected (as determined by a synthetic CAGE uniqueness track, see Methods) distribution of CAGE-inferred TSS locations (vertical axis) +/- 500 bp upstream/downstream and across the body of major TE classes (horizontal axis). TSS locations are visualized separately for the sense (upper panel) and antisense (lower panel) strands.*

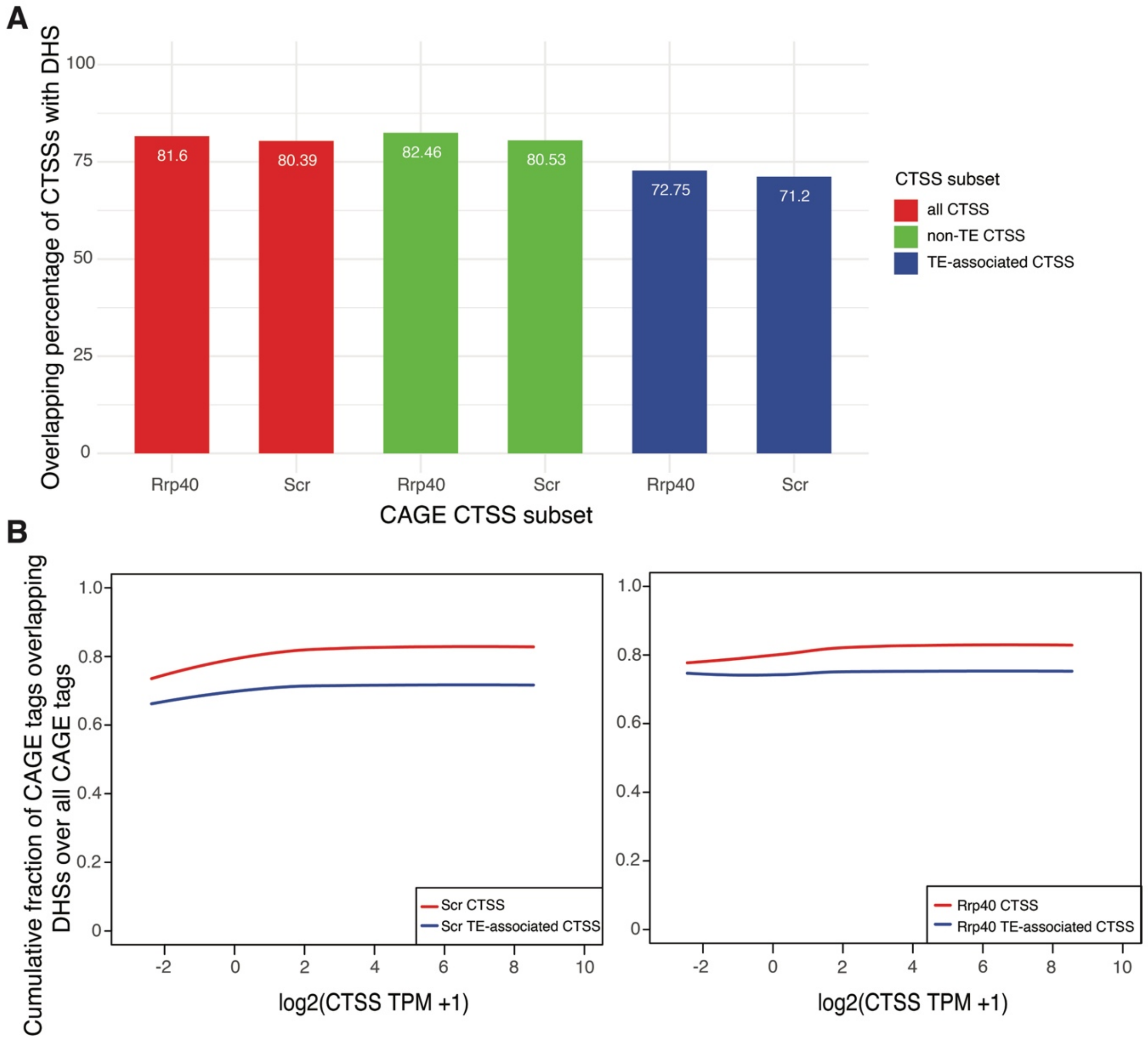

***Supplementary Figure 7**. **A:** Percentage of CAGE CTSSs within +/- 250 bp of DHS midpoints out of total in each of the subsets: all called (red), all non-TE associated (green) and all TE-associated (blue) CAGE CTSS in control and exosome-depleted CAGE libraries, respectively. **B:** Cumulative fraction of CAGE tags within +/- 250 bp of DHS midpoints out of total CAGE ctss in control (Scr, left panel) and exosome-depleted (Rrp40, right panel) CAGE libraries versus CTSS expression level (log_2-transformed TPM-normalized counts). The cumulative fraction for all CAGE CTSS (red) and TE-associated CTSSs (blue) is shown.*

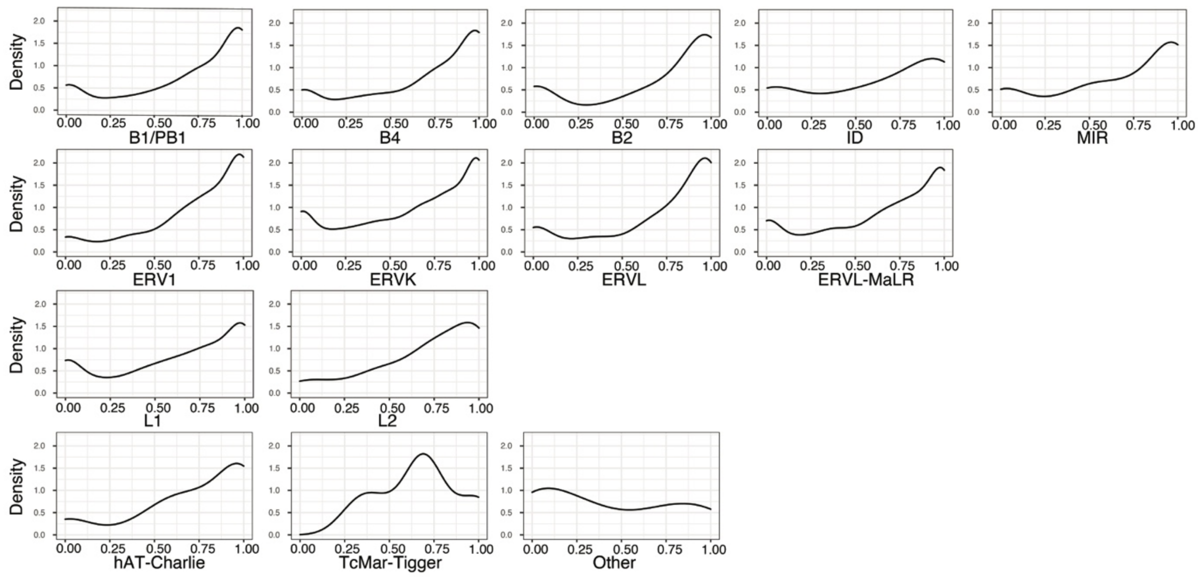

Supplementary Figure 8*. Densities of exosome sensitivity score, measuring the relative amount of exosome degraded RNAs, for transcripts associated with major TE families based on DHS-associated strand-specific expression levels in control and exosome-depleted CAGE libraries. The exosome sensitivity score ranges from 0 (fully captured by control CAGE) to 1 (only observed upon exosome depletion).*

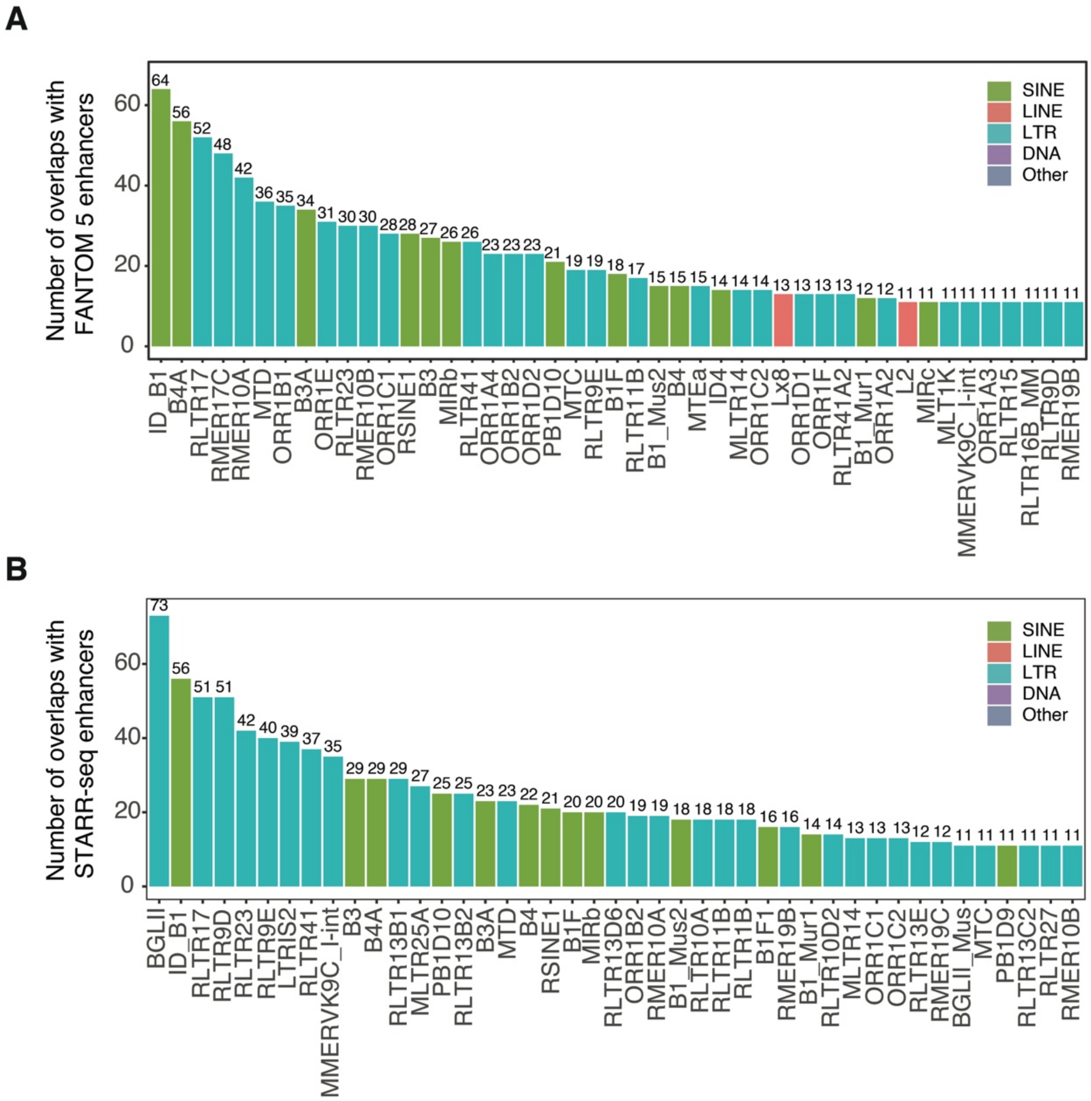

Supplementary Figure 9. *The number of transcribed TEs overlapping FANTOM5 mouse enhancers (A) and STARR-seq mESC enhancers (B) at the TE subfamily level.*

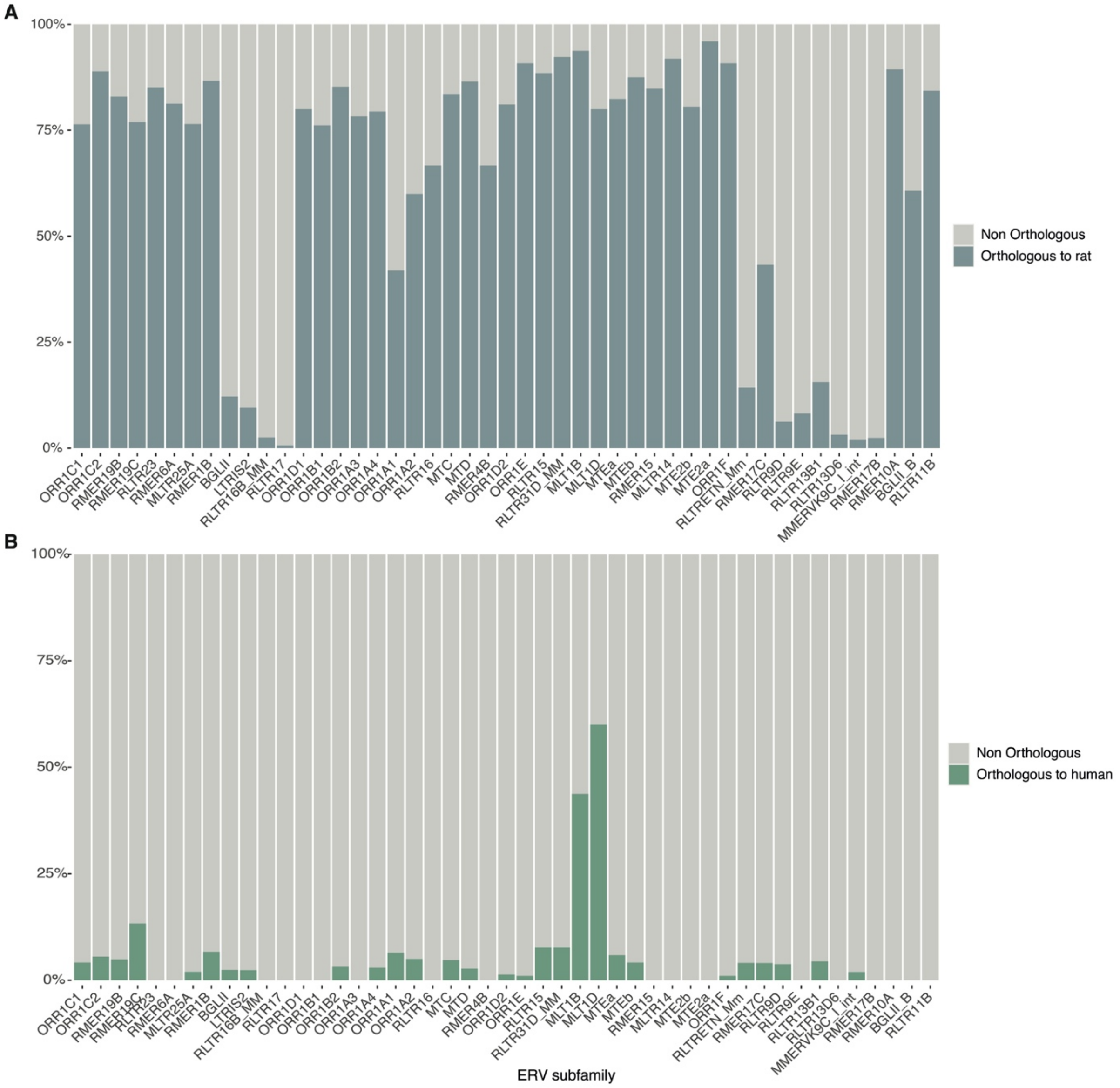

Supplementary Figure 10: *Proportion of mouse TE-associated expressed DHSs with orthologous regions in the rat (A) and the human (B) genomes split by ERV subfamily.*

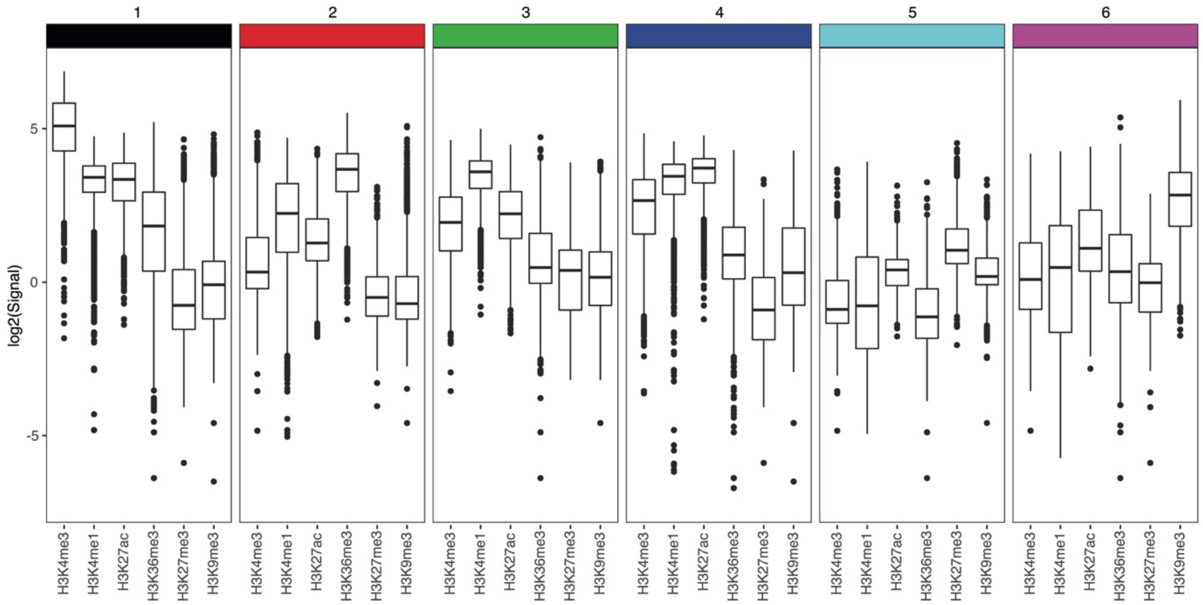

***Supplementary Figure 11**: Box-and-whisker plots of log_2-transformed histone modification (ChiP-seq) signal around summits of TE-associated clusters of CAGE-inferred TSSs for groups identified by hierarchical clustering, as shown in Figure 4A. Central band: median; boundaries: first and third quartiles; whiskers: +/- 1.5 IQR.*

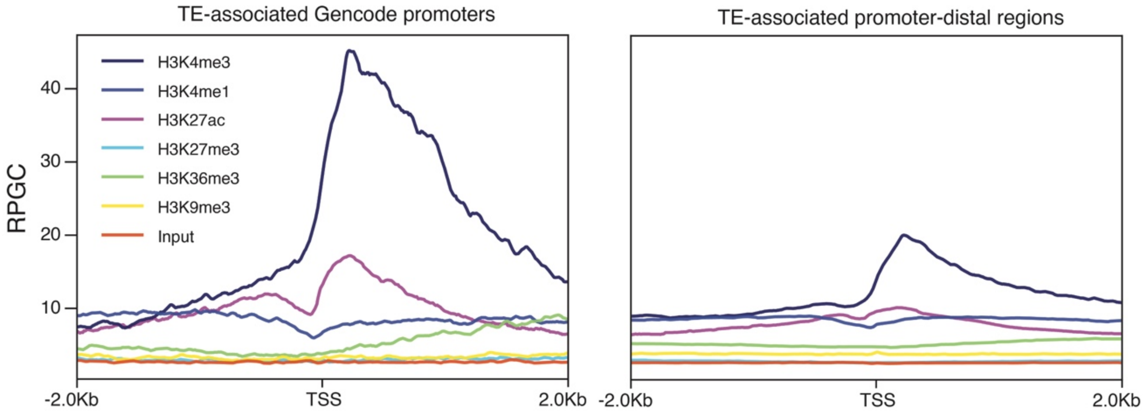

***Supplementary Figure 12**: Average ChIP-seq signal for histone modifications (H3K4me3, H3K4me1, H3K27ac, H3K27me3, H3K36me3, H3K9me3) and input +/- 2 kb around the summits of TE-associated CAGE-inferred TSS clusters at GENCODE promoters (left) and at gene-distal locations (right). Signals shown are reads per genomic context (RPGC), normalized for sequencing depth to 1x genome coverage.*

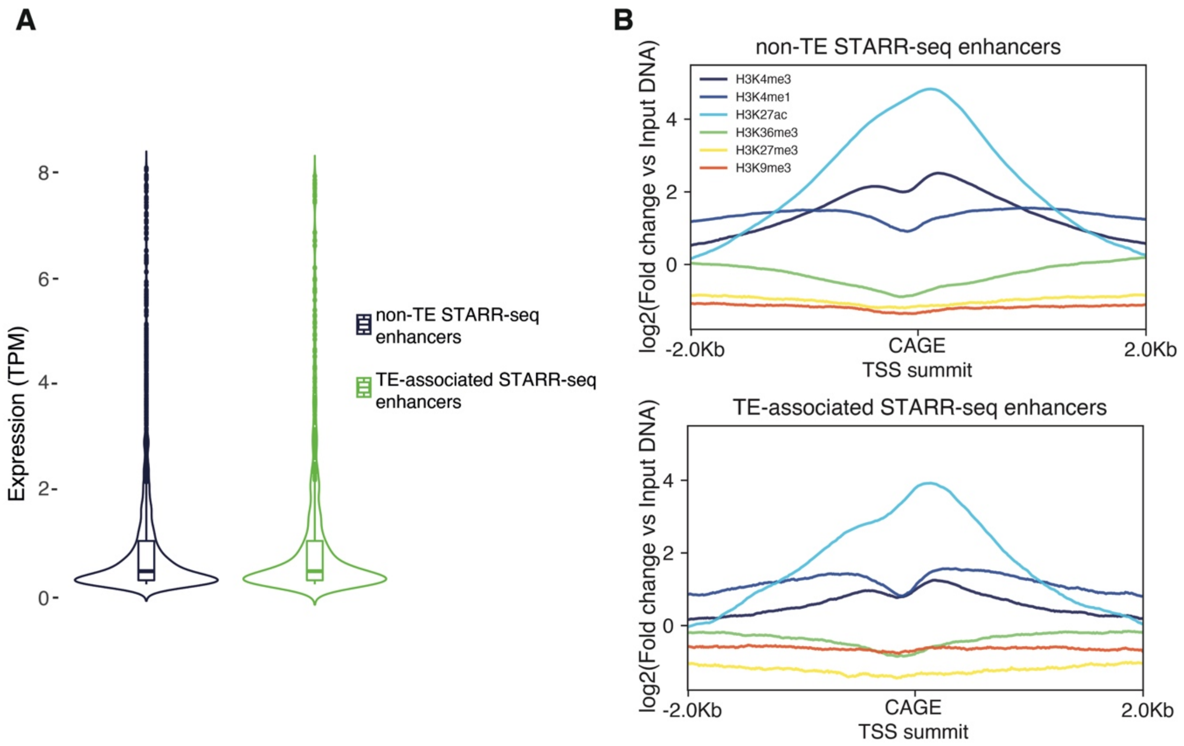

***Supplementary Figure 13**. **A:** TPM-normalized expression of TE-associated and non-TE-associated CAGE-inferred TSS clusters (TCs) at STARR-seq-associated DHSs. **B:** average normalized ChiP-seq signal for histone modifications (fold change over input DNA) for transcribed STARR-seq-associated DHSs not associated (top) or associated (bottom) with TEs.*

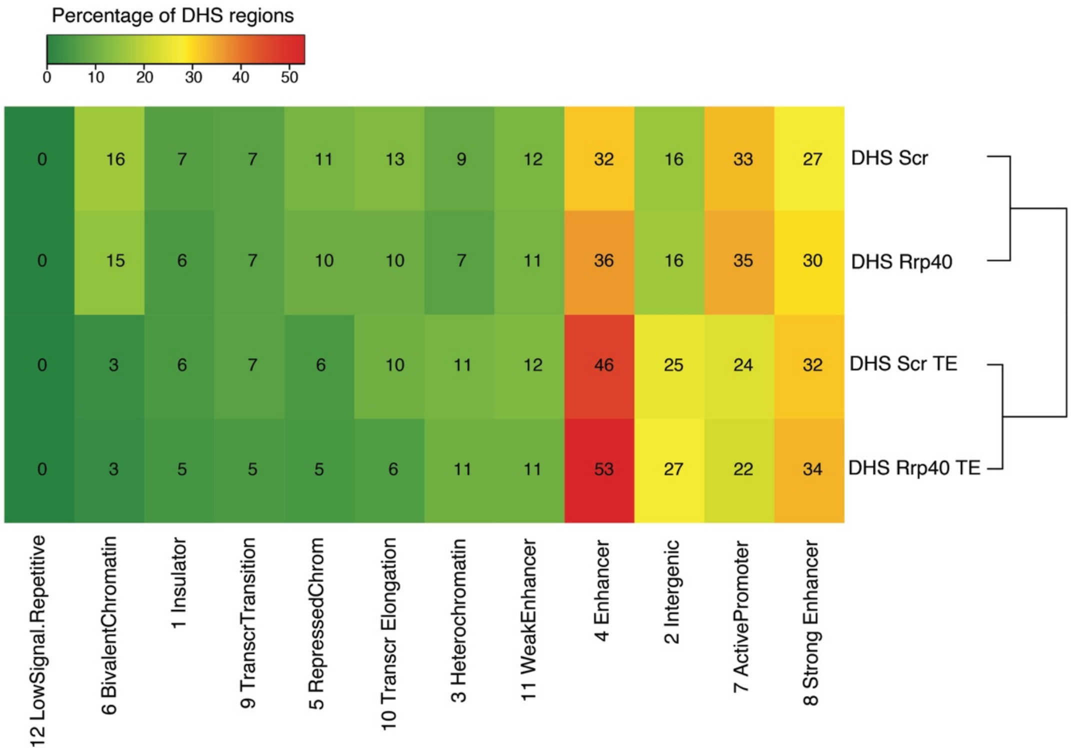

Supplementary Figure 14. 12-state ChromHMM model revealing the enrichment of specific combinations of histone modifications and TF marks at expressed DHS-associated genomic regions. The color key (from green (low) to red (high)) and the values in the heatmap cells show the percentage of DHSs that overlap each different ChromHMM state (horizontal axis) that are either expressed in control (Scr) and exosome-depleted (Rrp40) CAGE libraries, split by considering either all expressed DHSs or those that also overlap transcribed TEs.

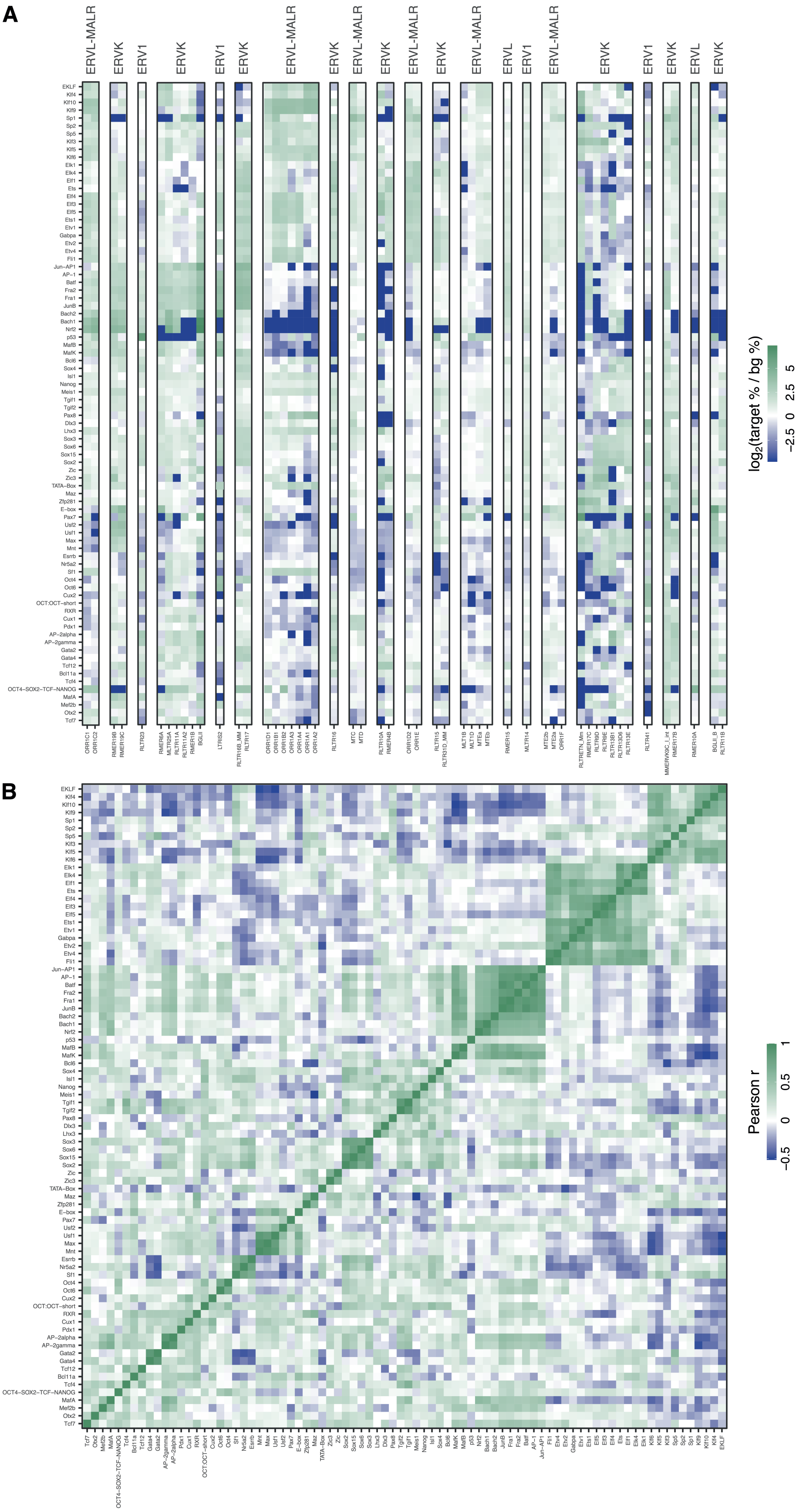
A
B
ERVL-MALR
ERVK
ERV1
ERVL
log2(target % / bg %)
Pearson r

Supplementary Figure 15. A: *Motif enrichments for TFs (columns) in TE-associated transcribed DHSs for all ERV subfamilies (rows) versus a background of genomic regions +/- 200 bp around the summits of all CAGE-inferred TSS clusters. White cells indicate no enrichment and cases of complete depletion were assigned the lowest detected score, represented in dark blue according to the scale.* ***B:*** *Pearson's correlation coefficients for pairwise TF motif co-occurence across all ERV subfamilies.*

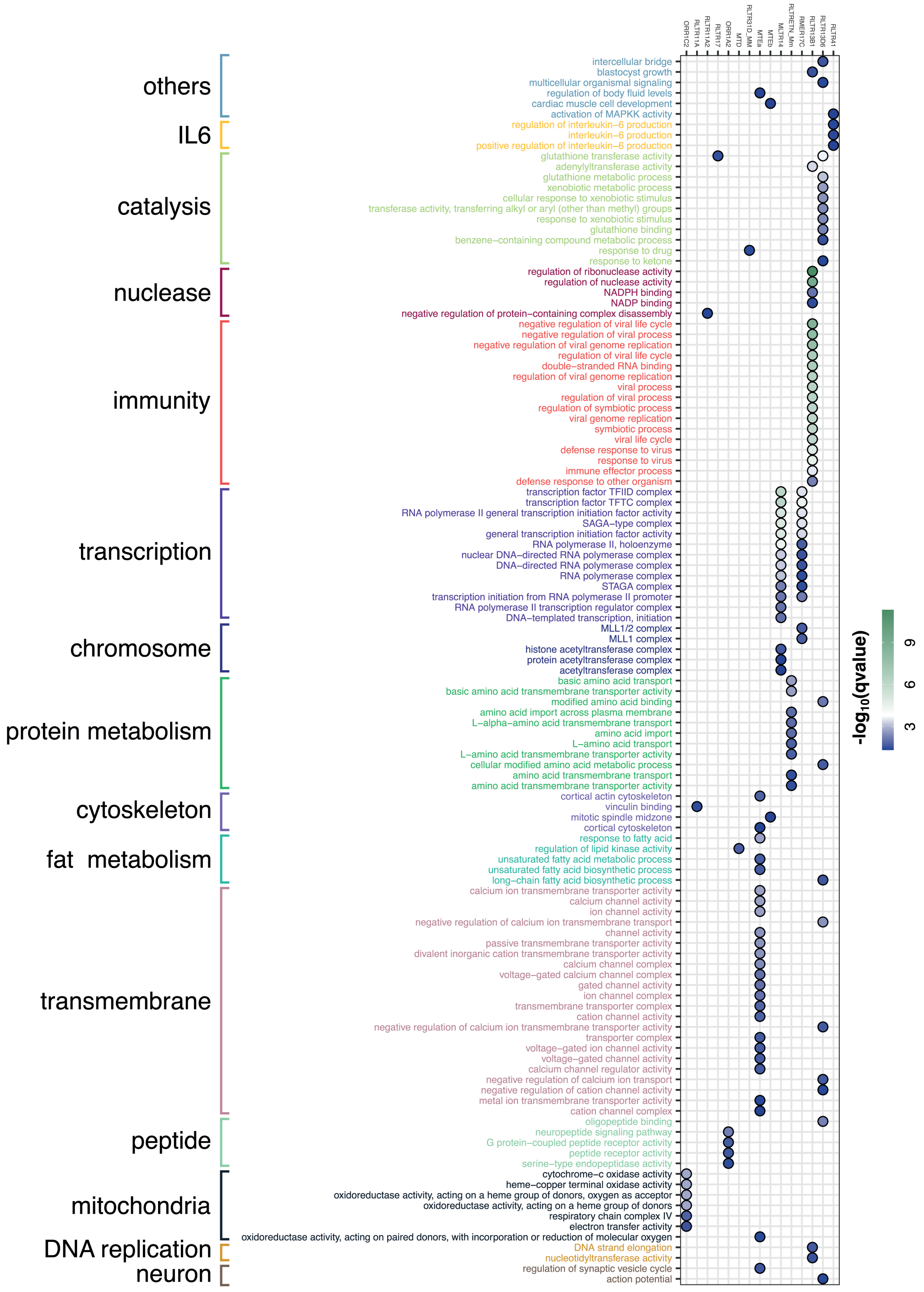

Supplementary Figure 16. *Enriched GO terms based on putative target genes (ABC) of gene distal ERVs for individual ERV subfamilies (foreground, rows) versus all ABC-predicted target genes (background). All enriched GO terms are manually curated into processes or functions and colored accordingly. Points are colored according to the transformed q-values indicating the significance of the GO-term enrichment.*
