# Supplementary material for: Endogenous retroviruses co-opted as divergently transcribed regulatory elements shape the regulatory landscape of embryonic stem cells
Source: Nucleic Acids Res. 2022 Feb 15;50(4):2111–27. doi: 10.1093/nar/gkac088 (PMC8887488; doi:10.1093/nar/gkac088)
Supplement: gkac088_Supplemental_Files [file gkac088_supplemental_files.zip › Supplementary_Tables_Legends.docx]

**Supplementary Tables**

**Supplementary Table 1: Average TPM-normalized expression values for major TE subfamilies in mESCs**

Row names: major TE subfamilies (with over 50 expressed instances) as annotated by RepeatMasker.

Columns:

- TPM Scr Unique: Average TPM expression quantified using only uniquely mappable reads from control CAGE libraries
- TPM Scr MumRescued: Average TPM expression quantified from control CAGE libraries using uniquely mappable and rescued reads after employing the MumRescueLite algorithm
- TPM Rrp40 Unique: Average TPM expression quantified using only uniquely mappable reads from exosome-depleted CAGE libraries
- TPM Rrp40 MumRescued: Average TPM expression quantified from exosome-depleted CAGE libraries using uniquely mappable and rescued reads after employing the MumRescueLite algorithm

**Supplementary Table 2: Matrix of log_2_-transformed motif enrichment scores per LTR subfamily**

Values correspond to % of TE-associated transcribed DHSs with motif / % of background sequences with motif. Background sequences were extracted +/- 200 bp around the summits of all CAGE-inferred TSS clusters. -Inf values (complete depletion from foreground) are provided as “minus_Inf”.

Row names: transcription factor motif names

Columns: LTR subfamily names as annotated by RepeatMasker
